# Can the Hemoglobin-Albumin-Lymphocyte-Platelet (HALP) Index Be Used as a Prognostic Marker in Patients Diagnosed With Idiopathic Pulmonary Fibrosis?

**DOI:** 10.7759/cureus.88423

**Published:** 2025-07-21

**Authors:** Esma Sevil Akkurt, Kerem Ensarioglu, Berna Akıncı Ozyurek, Tugce Sahin Ozdemirel, Ozlem Duvenci Birben, Tuhanan Dolmuş, Ozlem Ertan

**Affiliations:** 1 Pulmonary Medicine, University of Health Sciences, Ankara Atatürk Sanatorium Training and Research Hospital, Ankara, TUR; 2 Pulmonary Medicine, University of Health Sciences Ankara Atatürk Sanatorium Training and Research Hospital, Ankara, TUR; 3 Pulmonary Medicine, University of Health Sciences, Ankara Oncology Training and Research Hospital, Ankara, TUR

**Keywords:** halp score, idiopathic pulmonary fibrosis, prognostic marker, systemic inflammation, usual interstitial pneumonia (uip)

## Abstract

Background: The hemoglobin-albumin-lymphocyte-platelet (HALP) score serves as an indicator of systemic inflammation and can be utilized as a prognostic marker. It has been previously associated with many cancers, but its relationship with prognosis in patients diagnosed with idiopathic pulmonary fibrosis (IPF) is unknown. In our study, we aimed to evaluate the usability of the HALP score as a prognostic marker in patients diagnosed with IPF.

Methods: The study evaluated patients with IPF who were diagnosed and had follow-up visits in the pulmonary medicine clinic of a tertiary hospital between January 2021 and March 2023. Patients’ clinical information, comorbidities, laboratory values, pulmonary function test-diffusion capacity of the lung for carbon monoxide (PFT-DLCO) parameters at the time of diagnosis, six-minute walk tests, body mass indices, prognosis information, and death information were recorded.

Results: A total of 224 patients were included in the study, with a mean age of 66.27 (±8.83) years and a predominantly male population (84%). The mean HALP score was 53.27 (±27.73), and with a cut-off of 25 for HALP, survival duration of patients between groups were statistically; a median survival of 493 (40-945) days for patients with a score of 25 or below for HALP and 981 (752-1209) days for those above it (log-rank test result of 0.01 and X^2^(1)= 6.693). Due to patient distribution, further subgroup evaluation could not be performed.

Conclusion: The HALP score is a reliable, simple, easily accessible, and inexpensive index for predicting the prognosis of IPF. Further studies are required to evaluate subgroup outcomes for IPF patients.

## Introduction

Idiopathic pulmonary fibrosis (IPF) is a chronic and progressive lung disease characterised by fibrosis, with an unknown cause and a poor prognosis. In the presence of a typical clinical presentation (>50 years of age, insidious-onset progressive dyspnoea and dry cough), with a typical usual interstitial pneumonia (UIP) pattern on high-resolution computed tomography (HRCT), IPF can be clinically diagnosed with a multidisciplinary approach, if other possible causes of UIP are excluded [1]. Early diagnosis of IPF is important, since the survival time is around three to five years after diagnosis; however, the diagnosis is often delayed [2].

IPF patients must be evaluated at regular intervals in experienced centres. There is no single test that provides adequate prognostic information that can also be used to guide treatment decisions. Therefore, a comprehensive evaluation with clinical, morphological, and physiological parameters is required. Respiratory function tests, pulmonary exercise tests, effects on right heart functions, and radiological and histopathological patterns are among the important indicators in determining the prognosis of IPF [3]. Scoring systems of varying methods have been proposed as a means of evaluating IPF prognosis.

The hemoglobin-albumin-lymphocyte-platelet (HALP) score consists of patients’ hemoglobin, albumin, lymphocyte, and platelet values. It serves as an indicator of systemic inflammation and can be utilized as a prognostic marker. The HALP score is calculated as hemoglobin (g/L) × albumin (g/L) levels × lymphocyte count (/L)/platelet count (/L). These parameters can be calculated based simply on the laboratory parameters of patients as used in daily practice. Studies have reported that the HALP score is a good prognostic marker for various types of cancer, including gastrointestinal and genitourinary cancers [4-6]. Although IPF is primarily a fibrotic disease, recent studies suggest that low-grade systemic inflammation may contribute to its progression. HALP, a composite score involving hemoglobin, albumin, lymphocytes, and platelets, may reflect the systemic physiological state of these patients.

The HALP score has been previously associated with various cancers; however, its relationship with prognosis in patients diagnosed with IPF remains unknown. In our study, we aimed to evaluate the usability of the HALP score as a prognostic marker in patients diagnosed with IPF.

## Materials and methods

The study included patients with IPF who were diagnosed and had follow-up visits in the pulmonary medicine clinic of our hospital between January 2022 and March 2024. Inclusion criteria were being over 50 years of age, being diagnosed with IPF according to ATS/ERS/JRS/ALAT Clinical Practice Guideline criteria, and having demographic information, comorbidity information, laboratory values, respiratory function test-diffusion capacity of the lung for carbon monoxide (PFT-DLCO) data, and six-month prognosis information available from the hospital system and/or patient records. Exclusion criteria included the presence of multiple comorbidities (two or more), malignancy, or active infection.

The patients’ clinical information, comorbidities, laboratory values, PFT-DLCO parameters at the time of diagnosis, six-minute walk tests, body mass indices, prognosis information, and death information were recorded. The majority of the patients included in this study were receiving antifibrotic therapy at the time of data collection. Specifically, 77.2% were treated with pirfenidone and 41.1% with nintedanib. The hospital’s ethics committee (No:2012-KAEK-15/2852) approved the study. This research was conducted in accordance with the Helsinki Declaration (as revised in 2013).

The HALP cut-off value of 25 was determined using receiver operating characteristic (ROC) analysis in our study population and is consistent with values reported in prior studies involving chronic pulmonary and oncologic conditions. Survival data were obtained through both hospital records and the national death registry. Patients’ data were collected at the time of diagnosis, and those with acute exacerbations were excluded.

We excluded patients with more than two chronic comorbidities to reduce confounding, although residual bias may remain. Serial pulmonary function data were not available; hence, this study focused on baseline HALP assessment only. Although most individual HALP components were within normal ranges, the aggregate score still stratified survival.

Statistical evaluation

The patients’ results were put into a Microsoft Excel (Microsoft® Corp., Redmond, WA) file for overall evaluation. After investigating any mis-input and values, the data were moved to a statistics module (Statistical Product and Service Solutions (SPSS, version 25; IBM SPSS Statistics for Windows, Armonk, NY). The initial assessment was performed by descriptive analysis, for which values were given with mean and standard deviation or with median and percentiles as required. Parametric distribution was evaluated by Q-Q plot analysis. Independent sample T-test comparisons were made between groups for parametric values, while the Mann-Whitney U test was used for nonparametric values. ROC analysis was used to investigate the HALP score on survival at a given cut-off value. Kaplan-Meier survival analysis was used to compare overall survival between the two given HALP groups. Linear regression analysis was plotted to evaluate the role of additional parameters and to determine if a HALP grouping had a significant role in survival. It was decided that a p-value below 0.05 would be taken as statistically significant. The cut-off value for a HALP score was calculated from the available studies on pulmonary diseases regarding the role of HALP on mortality, which was later confirmed by the Kaplan-Meier survival log-rank test to be statistically relevant.

## Results

A total of 224 patients were included in the study group. The average age of the patients was 66.27 (±8.83) years, and the majority of patients (n=190, 84%) were male. The median duration of survival was 892 (286-1,520) days. A total of 161 patients (71.9%) were lost during the follow-up. Only two (0.9%) patients had required pathological diagnosis confirmation for IPF, and tomography findings were typical for UIP in most (n=172, 76.8%) of the patients. Chronic obstructive pulmonary disease was observed in 64 (28.6%) patients, followed by coronary arterial disease (n=51, 22.8%); these were the two most commonly reported comorbidities (Table 1).

**Table 1:** Demographic findings, diagnostic modalities, comorbidities, and survival duration

Parameters		Patient Count (n=224)
Age (mean, SD)		66.27 (8.83)
Survival duration (days, median, 25–75th)		892 (286-1520)
Gender (n,%)	Male	190 (84)
	Female	34 (15)
Diagnostic method (n,%)	Radiological	188 (83.9)
	Pathological	2 (0.9)
	Both	34 (15.2)
Chronic obstructive pulmonary disease (n,%)		64 (28.6)
Coronary arterial disease (n,%)		51 (22.8)
Hypertension (n,%)		41 (18.3)
Diabetes (n,%)		35 (15.6)
Congestive heart failure (n,%)		11 (4.9)
Pulmonary thromboembolism (n,%)		13 (5.8)
Cerebrovascular event (n,%)		2 (0.9)
SD: Standard deviation		

Pirfenidone was the predominant treatment modality chosen for the patients (n=173, 77.2%). At the time of diagnosis, the average forced expiratory volume 1 (FEV1), forced vital capacity (FVC), and DLCO were reported as 78.58% (±17.38), 72.97% (±16.26), and 57.24% (±20.78), respectively. Regarding laboratory parameters in the routine blood count, haemoglobin, lymphocyte, neutrophile and platelet were 14.3 (±1.8) g/dL, 2.23 (±0.94) G/L, 5.94 (±2.34) G/L, and 247 (±74) G/L, respectively. Total protein and albumin levels were within normal ranges, at reported values of 7.3 (±0.67) g/dL and 3.87 (±0.48) g/dL. The mean of the calculated HALP score was 53.27 (±27.73). All values, excluding survival days, had normal distribution patterns (Table 2).

**Table 2:** Respiratory function test, laboratory markers, and HALP results DLCO: Diffusion capacity of the lung for carbon monoxide; HALP: Hemoglobin-albumin-lymphocyte-platelet; VA: Alveolar ventilation; SD: Standard deviation

Parameters		Patient count (n=224)
Treatment history (n, %)	Pirfenidone	173 (77.2)
	Nintedanib	92 (41.1)
Forced expiratory volume 1 (%, SD)		78.58 (17.38)
Forced vital capacity (%, SD)		72.97 (16.26)
DLCO (%, SD)		57.24 (20.78)
DLCO/VA (%, SD)		79.62 (24.12)
Hemoglobin (g/dL, SD)		14.3 (1.8)
Total protein (g/dL, SD)		7.3 (0.67)
Albumin (g/dL, SD)		3.87 (0.48)
Lymphocyte (#, G/L, SD)		2.23 (0.94)
Platelet (#, G/L, SD)		247 (74)
Neutrophile (#, G/L, SD)		5.94 (2.34)
HALP Score (SD)		53.27 (27.73)

Patients were divided into two groups, with a HALP score cut-off of 25. When survival duration was compared between the two groups, it was statistically significant (721 vs. 934 days, p = 0.004). Respiratory function parameters did not change significantly between the two groups. Regarding the comorbidity comparison, patients with known chronic obstructive pulmonary disease (COPD) had an overall higher HALP score (56.44 vs. 45.63), which was statistically significant (p=0.010). In the case of coronary artery disease, the mean HALP values in both groups were similar, and there was no statistically significant difference (Table 3).

**Table 3:** Comparison of survival duration and respiratory function test results between HALP groups DLCO: Diffusion capacity of the lung for carbon monoxide; VA: Alveolar ventilation; SD: Standard deviation; T: Observed t value; df: Degrees of freedom; U: Mann-Whitney U statistic; Z: Standardized test statistic. *p < 0.05 was considered statistically significant.

Parameters	HALP < 25	HALP ≥ 25	p-value	T / U	df / Z
Survival duration (days, median [25–75th])	721 (63–984)	934 (341–1570)	0.004*	U = 326.5	Z = -0.304
Forced expiratory volume 1 (% ± SD)	80 ± 17	78.22 ± 17.34	0.754	T = 0.315	df = 72
Forced vital capacity (% ± SD)	72.66 ± 13.23	73.45 ± 16.53	0.876	T = -0.157	df = 85
DLCO (% ± SD)	59.61 ± 25.92	56.39 ± 19.94	0.605	T = 0.519	df = 94
DLCO/VA (% ± SD)	73.78 ± 27.28	80.53 ± 23.83	0.448	T = -0.765	df = 56

At the same cut-off value, the ROC analysis for survival days was performed, and the model was statistically significant, with an area under the curve (AUC) percentage of 67.3%, a standard error of 0.058, and a p-score of 0.004. Regarding survival analysis, the Kaplan-Meier model was statistically significant (a log-rank test result was 0.01 and X2(1)=6.693). The median survival for patients with a HALP score below 25 was 493 (40-945) days, while for those above 25, it was 981 (752-1,209) days.

A linear regression model could not be created, as scale parameters - including age, pulmonary function test results, and HALP scores - did not have a linear correlation with survival days. Heteroscedasticity was also present, which was more prominent in the analysis between survival days and the HALP score.

No significant differences were observed in baseline FVC or DLCO values between patients with HALP <25 and those with HALP ≥25. Although individual HALP parameters were mostly within normal ranges, the composite score still stratified survival outcomes. Although most individual HALP components were within normal ranges, the aggregate score still stratified survival.

## Discussion

The HALP score is a comprehensive index that measures a patient’s nutritional and immune health. For gastrointestinal cancer, oesophageal squamous-cell carcinoma, colorectal cancer, gastric cancer, and malignant tumors of the urinary system, the HALP score has been shown to have a prognostic impact [7-10]. However, no studies have investigated the HALP score’s prognostic importance in victims with idiopathic pulmonary fibrosis. Although the components of the HALP score are not specific indicators of pulmonary function or fibrosis extent, they reflect the overall inflammatory and nutritional status of the patient, which may contribute to prognosis in chronic diseases such as IPF.

In our study, when patients were divided into two groups based on a HALP score cut-off point of 25 and their survival times compared, a statistically significant difference was observed. Although the cut-off value was not derived from prospective validation, its statistical relevance in our survival analysis and its consistency with previous studies support its use as a provisional threshold. However, respiratory function parameters did not change significantly between the two groups. The median survival of patients with a HALP score below 25 was 493 (40-945) days, while it was 981 (752-1,209) days in those with a HALP score above 25. The cut-off value for the HALP score was calculated from the available studies on pulmonary diseases regarding the role of HALP on mortality, which was later confirmed by Kaplan-Meier Survival log-rank test to be statistically relevant. Upon examining the parameters used in the HALP score calculation, we noted a decrease in surveillance among patients with a score below 25, which led us to consider that the presence of anaemia and thrombocytosis may be indicators of a poor prognosis.

In a study conducted in Turkey, where the HALP score was used to predict prognosis in patients with advanced non-small cell lung cancer (NSCLC), 401 patients were included [5]. In the ROC curve analysis, the cut-off point was found to be 23.24 for HALP. Patients were divided into low and high HALP groups according to these cut-off points. The patients had a median HALP score of 29.08. Men with a high Eastern Cooperative Oncology Group (ECOG) score and progressive disease after first-line, platinum-based chemotherapy were found to be associated with a low HALP score. The HALP score showed no differences based on age, history of smoking, BMI, tumour histology, or chemotherapy regimen. Groups with lower HALP scores had significantly shorter overall survival (OS) compared to those with higher HALP scores. In our study, patients were divided into two groups with a HALP score cut-off of 25. When survival duration was compared between the two groups, it was statistically different, but all values, excluding survival days, had normal distribution patterns.

In the study conducted by Zhai et al. in 2021, which examined the analytical value of the HALP score and lymphocyte-monocyte ratio (LMR) after radical lung cancer surgery in patients with NSCLC, 238 patients diagnosed with NSCLC were retrospectively examined [11]. The Kaplan-Meier survival assessment showed that the OS of the highest HALP class was significantly higher than that of the lower HALP class, and the OS of the high LMR group was also revealed to be higher than that of the lower LMR class. Zhai et al. showed that preoperative HALP and LMR were predictive of NSCLC in patients after radical lung cancer surgery and were independent risk factors for predicting NSCLC in the study. Similar to their study, our study showed that the overall survival rate of the group with a high HALP score was significantly higher than the group with a low HALP score.

A cohort study was conducted to investigate the relationship between HALP scores and the risk of ICU mortality in patients with acute exacerbation of COPD (AECOPD), involving 1,533 patients. Low HALP scores and low LMR scores were associated with an increased ICU mortality risk in patients with AECOPD, after adjusting for all confounders. A low HALP score was associated with an increased risk of intensive-care mortality in patients with AECOPD [12]. As a result of that study, it was suggested that the HALP score could serve as a new prognostic marker in patients with AECOPD. Our study supports a similar assumption that the HALP score could be a new prognostic marker in IPF patients as well as in AECOPD patients. Although albumin is not a disease-specific biomarker for IPF, it reflects the systemic nutritional and inflammatory milieu. Therefore, its inclusion in the HALP score aligns with prior research across multiple chronic diseases and preserves scoring consistency.

A single-center study investigating the effect of bronchiectasis exacerbation on the HALP score found that the HALP score was associated with infectious and pulmonary functional parameters in bronchiectasis patients during the exacerbation period. While the HALP score was found to be significantly lower in patients in the exacerbation period compared to the stable period, a positive, significant relationship was found between the HALP score and FEV1% and FVC% [13]. In our study, survival times were statistically different between the two groups; however, no significant changes in respiratory function parameters were observed between the two groups. A linear regression model could not be created, as scale parameters - including age, pulmonary function test results, and HALP scores - did not have a linear correlation with survival days.

A study by Mutlu et al. [14] of 115 patients aimed to increase understanding of how variables such as nutritional status and inflammation indices affect the progression and prognosis of malignant mesothelioma in patients. While pre-treatment high neutrophil/lymphocyte ratio (NLR), high pan-immune inflammation value (PIIV), and high systemic inflammation response index (SIRI) were associated with high advanced lung cancer inflammation index (ALI), a high HALP score has been found to be associated with favourable survival [14]. In our study, the median survival for patients with a HALP score below 25 was 493 (40-945) days, while for those above 25, it was 981 (752-1,209) days; this was statistically significant.

The prognosis of patients with IPF is severe, with an average survival of approximately three years. A reduction in forced vital capacity or diffusion capacity of the lung for carbon monoxide (DLCO) and acute exacerbation may be important risk factors for survival with IPF. Two fibrotic drugs, nintedanib and pirfenidone, appear to be safe and effective for IPF. A meta-analysis of eight randomised controlled and 18 cohort studies in 12,956 patients with IPF showed a reduced risk of all-cause mortality and acute exacerbations with antifibrotic therapy, including nintedanib and pirfenidone. However, determining the appropriate continuation of recovery from IPF is challenging because the rate of disease progression varies among individuals. Therefore, prognostic factors are required to decide treatment methods for IPF. However, there is no established biomarker to predict death, progression, or response to therapy in individuals with IPF. In previous reports, gender-age-pulmonary physiology (GAP) stage based on clinical (e.g., gender or age) and apparent (e.g., FVC and DLCO) variables could predict overall survival in IPF patients. Studies show that patient-related outcomes (mortality, hospitalization, and costs) do not differ between the two currently available antifibrotic drugs, pirfenidone and nintedanib [15-17]. Thus, the decision between treatment with pirfenidone and treatment with nintedanib should be made on a case-by-case basis, taking into account clinical features, comorbidities, concomitant medications, side-effect risks, and patient preferences. In our study, pirfenidone was the most commonly chosen treatment modality for patients (77.2%). Although most patients use pirfenidone, we believe that a high baseline HALP score is indicative of a good prognosis, regardless of the medication.

Although individual components such as hemoglobin, albumin, lymphocytes, and platelets were within normal ranges in many cases, their combination into the HALP index allowed for effective prognostic differentiation, underscoring the value of composite scoring. Due to the retrospective design, multivariate analyses could not be performed, and the HALP score’s independence as a prognostic factor in IPF remains to be validated. We excluded patients with more than two significant comorbidities to reduce confounding, but the potential for residual bias remains. Furthermore, serial pulmonary function data were unavailable, limiting longitudinal assessment of disease progression.

Limitations

A limitation of this study is the lack of a regression model for survival-duration correlation with other parameters. This lack was attributed to the nonparametric distribution of the survival duration, its non-linear correlation with other parameters, and being evaluated for heteroscedasticity, which states an uneven distribution of variance of errors. The rather arbitrary cut-off given for the HALP score, while validated from currently available literature regarding respiratory comorbidities, was a limiting factor as well. In addition to ROC analysis for survival evaluation, a confirmation of the said analysis was performed by Kaplan-Meier for validation purposes. However, a more varied population could have presented a different and more reliable cut-off analysis. Subdivision of survival for different parameters, including gender or treatment regimens, also could not be given due to the mentioned limitations.

Although most patients in our cohort were receiving antifibrotic therapy, we did not perform subgroup analysis based on treatment type. Therefore, we acknowledge that treatment status could have influenced survival outcomes and represents a limitation of our study.

Another limitation could be stated that, to evaluate HALP as a score for IPF patients, patients with more than two comorbidities that could have affected HALP scoring were excluded from the study. This had created a selection bias as per the nature of the study and could have been avoided only if a patient pool larger than what was available for a rare disease such as IPF had been available.

The retrospective design of our study precluded multivariate analysis; therefore, the HALP score’s independent prognostic value could not be confirmed. Baseline HALP values may offer prognostic value, but longitudinal changes could better capture disease progression dynamics. Therefore, future studies should evaluate serial HALP measurements in relation to clinical outcomes.

## Conclusions

Despite its limitations, our findings suggest that the HALP score - a reliable, simple, easily accessible, and inexpensive biomarker - may offer prognostic information in patients with IPF. Although it has primarily been studied in patients with cancer or other inflammatory diseases, we believe that combining the HALP score with other indices in future prospective, multivariate, and longitudinal studies could provide more specific insights into disease prognosis, including in IPF.

## References

[1] Raghu G, Remy-Jardin M, Richeldi L (2022). Idiopathic pulmonary fibrosis (an update) and progressive pulmonary fibrosis in adults: an official ATS/ERS/JRS/ALAT clinical practice guideline. Am J Respir Crit Care Med.

[2] Ley B, Bradford WZ, Vittinghoff E, Weycker D, du Bois RM, Collard HR (2016). Predictors of mortality poorly predict common measures of disease progression in idiopathic pulmonary fibrosis. Am J Respir Crit Care Med.

[3] Uzer F, Cilli A, Hanta I (2024). Assessment of quality of life in IPF Patients: a multicenter observational study. Sarcoidosis Vasc Diffuse Lung Dis.

[4] Yang N, Han X, Yu J, Shu W, Qiu F, Han J (2020). Hemoglobin, albumin, lymphocyte, and platelet score and neutrophil-to-lymphocyte ratio are novel significant prognostic factors for patients with small-cell lung cancer undergoing chemotherapy. J Cancer Res Ther.

[5] Güç ZG, Alacacıoğlu A, Kalender ME (2022). HALP score and GNRI: simple and easily accessible indexes for predicting prognosis in advanced stage NSCLC patients. The İzmir oncology group (IZOG) study. Front Nutr.

[6] Shen XB, Zhang YX, Wang W, Pan YY (2019). The hemoglobin, albumin, lymphocyte, and platelet (HALP) score in patients with small cell lung cancer before first-line treatment with etoposide and progression-free survival. Med Sci Monit.

[7] Cong L, Hu L (2017). The value of the combination of hemoglobin, albumin, lymphocyte and platelet in predicting platinum-based chemoradiotherapy response in male patients with esophageal squamous cell carcinoma. Int Immunopharmacol.

[8] Jiang H, Li H, Li A (2016). Preoperative combined hemoglobin, albumin, lymphocyte and platelet levels predict survival in patients with locally advanced colorectal cancer. Oncotarget.

[9] Chen XL, Xue L, Wang W (2015). Prognostic significance of the combination of preoperative hemoglobin, albumin, lymphocyte and platelet in patients with gastric carcinoma: a retrospective cohort study. Oncotarget.

[10] Peng D, Zhang CJ, Tang Q (2018). Prognostic significance of the combination of preoperative hemoglobin and albumin levels and lymphocyte and platelet counts (HALP) in patients with renal cell carcinoma after nephrectomy. BMC Urol.

[11] Zhai B, Chen J, Wu J (2021). Predictive value of the hemoglobin, albumin, lymphocyte, and platelet (halp) score and lymphocyte-to-monocyte ratio (lmr) in patients with non-small cell lung cancer after radical lung cancer surgery. Ann Transl Med.

[12] Han H, Hu S, Du J (2023). Predictive value of the hemoglobin-albumin-lymphocyte-platelet (HALP) index for ICU mortality in patients with acute exacerbations of chronic obstructive pulmonary disease (AECOPD). Intern Emerg Med.

[13] Çolak M, Çoban H, Sarioğlu N, Yumrukuz Şenel M, Erel F (2024). Could the HALP score serve as a biomarker of bronchiectasis exacerbation?. Tuberk Toraks.

[14] Mutlu E, Inanc M (2024). Prognostic significance of inflammation scores in malignant mesothelioma. Eur Rev Med Pharmacol Sci.

[15] Neighbors M, Cabanski CR, Ramalingam TR (2018). Prognostic and predictive biomarkers for patients with idiopathic pulmonary fibrosis treated with pirfenidone: post-hoc assessment of the capacity and ascend trials. Lancet Respir Med.

[16] Maher TM, Stowasser S, Nishioka Y (2019). Biomarkers of extracellular matrix turnover in patients with idiopathic pulmonary fibrosis given nintedanib (inmark study): a randomised, placebo-controlled study. Lancet Respir Med.

[17] Organ LA, Duggan AR, Oballa E (2019). Biomarkers of collagen synthesis predict progression in the PROFILE idiopathic pulmonary fibrosis cohort. Respir Res.

